# Opening the black box of quality improvement collaboratives: an Actor-Network theory approach

**DOI:** 10.1186/1472-6963-10-265

**Published:** 2010-09-08

**Authors:** Tineke Broer, Anna P Nieboer, Roland A Bal

**Affiliations:** 1 Department of Health Policy and Management, Erasmus University Rotterdam, Rotterdam, The Netherlands

## Abstract

**Background:**

Quality improvement collaboratives are often labeled as black boxes because effect studies usually do not describe exactly how the results were obtained. In this article we propose a way of opening such a black box, by taking up a dynamic perspective based on Actor-Network Theory. We thereby analyze how the problematisation process and the measurement practices are constructed. Findings from this analysis may have consequences for future evaluation studies of collaboratives.

**Methods:**

In an ethnographic design we probed two projects within a larger quality improvement collaborative on long term mental health care and care for the intellectually disabled. Ethnographic observations were made at nine national conferences. Furthermore we conducted six case studies involving participating teams. Additionally, we interviewed the two program leaders of the overall projects.

**Results:**

In one project the problematisation seemed to undergo a shift of focus away from the one suggested by the project leaders. In the other we observed multiple roles of the measurement instrument used. The instrument did not only measure effects of the improvement actions but also changed these actions and affected the actors involved.

**Conclusions:**

Effectiveness statistics ideally should be complemented with an analysis of the construction of the collaborative and the improvement practices. Effect studies of collaboratives could benefit from a mixed methods research design that combines quantitative and qualitative methods.

## Background

Ever since the US Institute of Medicine described the so-called "quality chasm" in health care [1], quality improvement has become an important policy issue. A proposed solution for bridging the chasm is setting quality improvement collaboratives (QIC's) to work. A nice example is the Breakthrough Series model that brings together teams from different hospitals or clinics with the aim to attain improvements on a certain theme [2]. The QIC model in general and BTS in particular are widely adopted in Western countries [3]. So far, there is little evidence, however, on the effectiveness of QIC's [3,4].

Despite the lack of evidence concerning effectiveness of QIC's, most studies evaluating QIC's are investigating their effectiveness rather than follow the collaborative as it gets formed. Bate and Robert argue that many evaluation studies take up an approach they describe as "summative, noninterventionist, and heavily reliant on quantitative assessments of "success"", which is "outcome-oriented" [5]. They contrast this approach to an action-oriented or formative approach that is mainly qualitative and that is devised to improve the method along the way by giving feedback to leaders of improvement projects. As (qualitative) process descriptions are lacking, QIC's are often described as "black boxes" [3,6]. Knowing what actually occurs in setting up and carrying out collaboratives would seem crucial for interpreting the effectiveness results [3,6,7].

Several suggestions for opening up the black box have been made. For example, Wilson et al asked collaborative leaders what they thought were crucial aspects of QIC's [6]. On the basis of the information retrieved they proposed a framework of core elements that have to be described in order to meaningfully link effectiveness data to the workings of the collaborative. This framework is limited, however, in that the set topic and main elements are considered to stay fixed during the project, as if it is just a matter of implementing the elements rather than the elements changing themselves as a result of implementation. By assuming that the topic of a QIC can be predefined, the authors for example do not focus on the construction of the QIC and also do not explore whether the topic may change during the collaborative process.

Bate and Robert and colleagues [8,9] took up a more dynamic approach by providing process descriptions of two collaboratives and detailing the extent to which the collaborative method was implemented. Yet also they did not analyze the way the features can be created or constructed within collaboratives. For example, they described the difficulties that measuring could pose for improvement teams, but they did not analyze how measurability was constructed or what different functions measuring could have within such projects [10]. They mainly looked at the success of implementation. In this sense, even Bate and Robert in their more dynamic approach still assume to know what collaboratives are before they even start opening the black box of collaboratives.

One of the reasons why this black box should be opened is to gain insight into the construction of effectiveness data within collaboratives, i.e. the relation between the topic and the outcomes of a QIC. As effectiveness is based both on the interventions carried out and on the way improvements are measured, two interrelated questions must be addressed. First, as summative research investigates the predefined effectiveness of a collaborative derived from the topic, it should be analyzed how this topic is created. Leading questions then concern possible changes in the topic during the project and the possible consequences of these changes for the predefined effectiveness. By focusing on the construction of the topic rather than on predefined elements, more insight is gained in what is actually done within the collaborative.

Secondly, the effectiveness measurement practices themselves should be analyzed. Within Breakthrough projects, measuring is assumed to play an important role. First, it helps in investigating a project's overall effectiveness. Second, teams themselves often use measurement instruments to investigate their own effectiveness and to adjust their improvement actions based on the results. But what roles do these measurement practices exactly play within QIC's and what is the relation between the topic and its measurability? Do the measurement instruments merely describe the topic and the improvements attained or do they affect the improvement practices as well?

In this article we will propose a way of opening the black box of collaboratives by using a dynamic perspective, though different from that of Bate and Robert. We study how the collaborative gets formed rather than taking fixed elements or the extent to which the elements are implemented as a starting point. To do so, we draw upon empirical material of two projects aimed at improving mental health care and care for the intellectually disabled. We studied these projects in the context of a larger evaluation study of the QIC they are part of.

The aim of our approach is threefold. First, we would like to propose a way for opening the black box of QIC's by focusing on their construction. Our second aim is to provide more insight into the dynamics of the collaborative process. So whereas the first question is more methodological, the second is an empirical question. Thirdly, we will study possible consequences of findings from this analysis for future evaluation studies.

The article is structured as follows. First we will describe a theoretical framework based on Actor-Network Theory (ANT). Then we will describe the two improvement projects and the way we gathered data for this paper. In the next sections we explore empirically what opening the black box of QIC's may mean from an ANT perspective. We do so by focusing on the way the topic and its measurability get constructed within the collaborative. In the conclusion we come back to the question what our analysis can add to (discussions on) evaluation studies of QIC's.

### Theoretical framework

As a methodology for opening the black box of QIC's, we draw on Actor-Network Theory. From an ANT perspective, none of a collaborative's elements is fixed before start of a project. Seen from this perspective a collaborative is a dynamic process in which its elements get constructed [11]. In drawing on ANT, researchers need to "follow the actors" and to analyze how these actors themselves define what is going on [12,13]. Therefore, we will not predefine the concept of 'collaborative' and its elements. Rather we look at the way the collaborative is formed during the project and what consequences this process has for the actors involved.

To analyze the dynamics within the topic of a collaborative, we use the ANT-notion of problematisation [14], which involves a dynamic way of defining and constructing the problem. Hence, seen from this perspective a problem is not given and already out there, but is constructed in a process in which actors can always (implicitly or explicitly) oppose the problematisation process. We use the term 'problematisation' instead of problem definition for it offers two advantages. First, it means that the problem definition emerges from a performance and not just from a perspective [15]. Secondly, it implies that the problematisation is not a singular event but is done over and over again, because (dynamic) practices make up a problematisation. Thereby, the term 'problematisation' allows us to follow how the different actors involved construct the topic of a QIC. The term suggests to investigate the way leaders of improvement projects present the topic, the way improvement teams participating in the project discuss the topic and the way teams perform it within the care organizations.

Looking at the problematisation process within improvement projects has already proven to be relevant. For example, Zuiderent-Jerak et al [16] showed that different problematisations can co-exist within a medication safety improvement project. In this project some teams focused on client autonomy whereas others sought to reduce medication errors. So the authors showed that teams may differ in doing a problematisation process, but they did not analyze the construction of the problematisation over the course of an improvement project, which will be our focus.

Next to studying the problematisation process, we investigate the measurement practices. From an ANT-perspective it is suggested not to make an a priori distinction between human and non-human actors [12]. The measurement instruments used within the projects can be perceived as non-human actors possibly contributing to the collaborative and the way the topic is performed. As said, measuring is assumed to play a dominant role in QIC's, notably regarding rapid cycle improvements. This means that improvement teams are to carry out small scale actions, measuring if the actions led to the expected outcomes, and, if not, adjusting the actions [2]. Furthermore, measuring is often used to estimate a project's overall effectiveness.

Yet ANT-scholars and other scholars have pointed at the performative effect of measurement instruments, meaning that instruments not only measure a situation but also affect this situation in foreseen and unforeseen ways. Perhaps the most famous example of performativity is that of opinion polls, which are aimed to investigate the expected election result but at the same time these polls influence actual voting behaviour and thus possibly change the election result. Furthermore, in social sciences measuring plays a profound role in shaping identities of persons and groups [17-19]. For example, Hacking holds that classification, including classification based on measuring, produces so-called looping effects, in which people react to the classification and make it either more true by behaving in line with the classification or make it less true by opposing to it [20]. So also here it is said that classification and measuring "interact" [20] with the world they refer to; they do not just represent this world but change it. As a last example, in organizational sciences Power [21,22] illustrated the performativity of measurements used within and between organizations. The data do not only represent practices in the organization but co-construct the organization and involved actors in foreseen and unforeseen ways. People sometimes start to focus mainly on the measures and attaining high results, thereby focusing less on other issues not captured in the measures [21,23].

Given that measurement practices can have a performative effect, their exact role(s) should be analyzed if we want to study the construction of a collaborative, because the measurement practices possibly affect the improvement practices and thereby also the performance of the topic - i.e. the problematisation - in foreseen and unforeseen ways. Consequently, if we want to address the question what effectiveness in improvement projects may mean, we should look at the way in which the problematisation process and the measurement practices are interlinked.

## Methods

### The collaborative approach

In this article we focus on two improvement projects that were part of a larger collaborative performed in the Netherlands: Care for Better. These projects were named 'recovery-oriented care' and 'social participation'. They aimed at improving long term mental health care and care for the intellectually disabled. Both projects started in 2007 and consisted of two rounds each lasting one year.

From twelve to fifteen improvement teams collaborated in each round of each project. Headed by a project leader, each team generally consisted of four to nine members. The two faculty teams of the projects consisted of an expert team and a core team made up of the program leader of the overall project and two or three 'process counsellors'. So in this article leaders of improvement teams are called project leaders, and the leaders of the overall project are called the program leaders. In the project 'recovery oriented care' only teams from mental health care participated. In the project 'social participation' a mixture of teams participated, some delivering care to intellectually disabled clients and others to psychiatric clients. The clients involved usually lived in a form of sheltered housing or at a ward of the institution.

For each round of each project, four national working conferences were organized at which faculty provided recommendations on the improvement actions and the method for improving. The starting conferences were mainly intended to familiarize teams with the proposed problem and improvement method. In the first and second working conferences the improvement practices were discussed in a mix of plenary sessions and workshops. The closing conference mostly served to sum up the results attained and to focus on sustaining and spreading the findings.

Despite the organizational similarities, the projects had different goals. The 'recovery-oriented care' project was devised to give clients more control over their lives, while the 'social participation' project aimed at enlarging and enriching the clients' social networks, supposedly making them feel less lonely. The exact interventions of improvement teams participating in these projects are part of our analysis and will be discussed in the results section.

### Research methods

We evaluated these projects within the context of our larger evaluation study of the Care for Better collaborative [16,24]. We carried out ethnographic observations at nine of the sixteen conferences, distributed over the two rounds of the two projects. Most data gathered at these conferences comprise lectures of the faculty team, reactions of improvement teams to these lectures, and discussions concerning the improvement practices. Furthermore, we separately interviewed the two program leaders. In addition, we studied six improvement teams in depth. These teams were selected on the grounds of observations of the conferences. In all cases we interviewed the project leaders of these improvement teams. Sometimes additional interviews with team members were conducted. These case studies usually lasted one day or one-half day.

We focus on only these two projects as it gives us the opportunity to analyze them more in depth, but other projects could be used as example as well. We draw on the 'recovery-oriented care' project to illustrate the problematisation process as suggested by faculty and actually performed by participating teams. In zooming in at the problematisation process, we first analyze the way faculty of the 'recovery-oriented care' project presented the problem and, interrelated, proposed solutions. Secondly, we analyze how teams discussed things and set improvement projects going [16], as well as whether and how they adopted the proposed problematisation of faculty. Measurement practices on the other hand are illustrated by observations from the 'social participation' project. We investigate the relation between the measurement practices and the initial problematisation of faculty, and furthermore explore the consequences of the measurement practices for the project and for the actors involved.

## Results

### The problematisation process

#### The initial problematisation: a lack of future perspective

The topic of the 'recovery-oriented care' project had been defined before the project went ahead. We entered stage at the starting conferences of the first round of the project, at which faculty introduced their ideas concerning problematisation. Their problematisation owed much to the recovery movement as initiated by former mental health care clients who described their recovery as a process of regaining control over their own lives, often leading to reintegration in society [25,26].

In line with the goals of the recovery movement, the main aim of the project was giving clients more space to govern their own lives and to make their own decisions. Many clients living in mental health care institutions were facing a bleak future, as faculty said. "The lives of clients within long term mental health care are characterized by routine, boredom, marginalization, and a lack of perspective," a former client who was part of the expert team said. As faculty saw it, one of the reasons for this situation was the "mental health care regime" in which clients are approached in a "stigmatizing" way or receive scant attention. This speaker added that many care givers do not have faith in the possibility of change in clients' conditions and skeptically asked "if this perception has changed now that mental health care has discovered the concept of recovery". "Do they [mental health care professionals] really believe that clients can [...] live a complete life?"

#### The proposed solution: reducing the role of professionals

Hence faculty defined the problem as follows: mental health care clients lack perspective owing to the way in which professionals approach them. Inherent to a problem definition is the question who should tackle the problem and what roles these actors should take up [27]. Interestingly, many teams in this project mainly consisted of professionals. However, through its focus on clients' recovery, the recovery movement does not define a very clear role for professionals [25,26].

Even faculty of this project struggled with this discrepancy. "It is an illusion to think that mental health care professionals can recover their clients," said one of the experts. The program leader moreover said that an improvement project is "a tricky thing" because it suggests that under certain conditions clear advances could be made within a year. The truth is we had no clear interventions available beforehand that would directly lead to clients' recovery, she said in an interview.

Still the faculty team had ideas for improvement. In order to support the clients' recovery process, professionals should be less dominantly involved in their lives. Ideally, as faculty said, professionals should restrict themselves to creating the essential preconditions for recovery to occur. For example, by removing those elements that are thought to be in the way of recovery, such as "restraining" home rules.

So this was how the faculty team saw the problem: many clients in mental health care institutions lack perspective because professionals do not show faith in their clients and do not give them much room for their wishes and plans. The improvement teams were therefore advised to step back.

#### The improvement actions: different ways of performing the project

Following faculty's problematisation process allowed us to analyze if and how the teams adopted this problematisation. Many teams recognized the picture sketched of clients lacking a future perspective. Also, they agreed they were too dominantly present in clients' lives. "Nurses often want to know everything, want to control everything, want to be in the lead," one project leader said. In an informal talk at a conference a team member said that while clients were often institutionalized, professionals were institutionalized as well.

So the teams seemed to adopt faculty's problematisation in the first instance. But how did they go ahead? As the recovery concept itself was seen as quite "abstract", many teams first set out to create a vision on what constituted recovery-oriented care. Furthermore, some teams discussed their approach to clients in line with the problematisation of faculty. One example concerned a client who changed clothes three times a day. Her bedroom door used to be locked to prevent her from doing so. The discussants wondered whether it was actually a problem that she changed clothes that often. They concluded it was not, unless this client was in "a manic period" and locking her door would calm her down. So this discussion indeed led to a proposal for reducing the professional role.

Another improvement action involved asking clients what hindered them in the way professionals approached them. One team invited clients to write down the home rules they disliked. This resulted in "a wall full" of post-its, said this team's project leader. One by one the post-its were taken from the wall and discussed. One client for example wanted a better arrangement for use of the washing machine. Another client disagreed with the lock on the refrigerator. This approach of asking clients what hinders them, in fact, is quite the reverse of the one in which professionals think up what might be hindering clients. It also has consequences for clients' role in their own recovery process; being either recipients or co-inventors of this new approach.

As another improvement action, clients were offered choice options in meals and snacks. "Despite being very psychotic, he is fully aware that he likes treacle wafers best," a team member said about a client who now chose treacle wafers every day. Yet many clients "do not know anymore what choosing means," a project leader said. One client, for example, even did not know what she would like to eat, although she expected the meals to be the best part of her holiday trip.

Many teams struggled with the question how to get clients to know and to state their wishes and how to get them into the recovery process, seeing that they "are often not easily mobilized and cannot mobilize and motivate themselves either", as a project leader told. "Some of my clients still think they are Napoleon," said another project leader to illustrate that clients may lack sense of reality. For these reasons, some team members told that recovery was not a suitable concept for their client group.

#### A change in problematisation: stimulating clients

These questions led to a change in problematisation. Improvement teams focused more on stimulating clients than on reducing their own roles in the lives of clients. The dilemma many team members faced was that they wanted to create a future perspective for clients but that clients themselves did not even have ideas about what they would like to eat, let alone what activities they wanted to undertake during their days or what life goals they had. Therefore some teams decided not to wait until clients could mobilize themselves, and to invent a program of activities themselves.

One team said that in the beginning of the project recovery looked like a figment to them. Clients could have been living for fourteen years within the institution and yet never have come up with the idea of breakfasting earlier than the set time, although they sometimes woke up at six o'clock. So the team proposed alternative meal times to clients. By the time of the closing conference these clients could have meals at variable times like in a hotel.

So during this project some teams shifted their focus from reducing their own role to stimulating clients. As faculty proposed it, the teams should strive for taking up as small a role as possible. However, some teams said that then nothing would happen and adopted a more active role in order to make clients more active as well.

#### Analysis of the problematisation process

To summarize, the 'recovery-oriented care' project was characterized by different problematisations. Faculty defined the main problem to be a lack of perspective for clients, and thought this was partly caused by a 'negative' approach from professionals. Faculty therefore advised teams to step back and to support the recovery process mainly by not hindering it. Some teams nevertheless took up a more dominant role in stimulating clients to become more active so as to improve their future perspective. This shift in the problematisation process also had consequences for "who has the right and who has the obligation" to do something about the problem [27], for example which actions from professionals were allowed and which actions were not.

So the problem on which an improvement project focuses may change during the course of the project. The exact problematisation depends on both the expert knowledge and the local knowledge of improvement teams. The improvement actions could not be directly deduced from the topic of the collaborative or from the way faculty proposed the problem, but had to be analyzed by following the actors.

The changes in problematisation were clearly notable in this improvement project because improvement teams were given much leeway. They had to state their own goals as a means to endorse the actions undertaken by them. At the same time faculty of the projects still tried to control the improvement actions by their own presentation of specific solutions. Another way of directing teams, intertwined with the problematisation process, is found in the measurement practice(s), illustrated in the next section by focusing on the 'social participation' project.

### The measurement practices

#### The 'social participation' improvement project

The two purposes of the 'social participation' project were to strengthen clients' social networks and, interrelated, to make them feel less lonely. Many clients have unfulfilled needs on the social domain, said the program leader at the starting conference. While he did some suggestions for improvement, he urged teams especially to adjust the improvement actions to the wishes and needs of clients themselves and to ask clients what they would like. The problematisation therefore was the following: although clients have many unfulfilled needs on the social domain, professionals do not always know and/or do not inform after these needs.

Consequently, many of the teams first set out to map the needs of clients, in order to see what improvements were possible in this regard. They often did so by using the network circle, which was an obligatory measurement instrument in this project and was meant to map all the contacts of clients. Options for improvement actions included contacting the persons important to clients or directing clients' attention to new contacts. For example, one client started to go to church; another regularly visited the sauna and there they met (new) people.

This project's central indicators for success were decided somewhere between the starting conference and the first working conference. The program leader proposed that social participation had "a subjective and an objective side", and teams were advised to direct their attention to either one of these pillars, or, ideally, to both. Teams were asked to measure both the subjective and the objective side at the beginning and the end of the project. The subjective side was measured in terms of the degree of loneliness clients experienced. The objective side was measured by the aforementioned network circle instrument, which is discussed in the next sections.

#### Shaping the actors: the assumptions within the instrument

The network circle is an instrument consisting of five concentric circles. The innermost represents the client himself or herself. The client's so-called anchors are placed in the next ring: "One can hardly imagine living without these people," one of the experts typified this circle. The third ring includes friends, "who enable you to do things you normally would not be doing". Then there are the acquaintances, "with whom you share one thing such as being part of the same tennis club". The outermost circle represents the professionals, who are getting paid to help clients. Professionals completed the network circle together with the client by informing after clients' contacts and where to place them. This approach was thought to bring up many unfulfilled needs and thus to open avenues for improvements aligned with clients' needs.

In making the network circle an obligatory measurement instrument, faculty assured that teams asked after clients' needs in this respect. The network circle in that sense was both an indicator of what faculty thought the problem and the solution were. The problem was, among other things, not enough information about clients' needs and/or the needs not being point of discussion. The solution accordingly was informing after these needs. Faculty could use the measurement instrument then to (subtly) steer the teams towards the proposed problematisation and solution.

Also in another way the measurement instrument supported the problematisation of faculty. Faculty said that professionals were often too dominantly present in a client's social life. Completing the network circle would tell them who else they could mobilize in order to improve the networks of clients. The instrument then would directly point at possibilities for reducing their role. Indeed, at the closing conference one of the experts said that this project was successful in that professionals had learned to abandon the notion that they were the ones who should manage everything for clients. So here again the measurement instrument had its function in strengthening the problematisation of faculty.

The instrument does not only carry assumptions about the professional role; it also "co-defined and co-produced" [17] the clients involved by assuming a typical client. Thereby, the instrument also stimulated professionals to assume this typical client and to approach clients in a certain way. For example, one of the assumptions in the instrument is that clients are able and interested to discuss their social network. Yet this was not always the case in this project. Some clients were reported to stay in bed the whole day, and their world accordingly was very restricted, as a project leader said.

Furthermore, clients were expected to be able to distinguish between professionals, friends and acquaintances. Yet many of the clients placed professionals in the friends ring. "From whose perspective do we fill this in?" a project leaders asked. "I have clients who designate my colleagues as their friends, is that allowed or not?" Faculty responded that clients should become aware that professionals cannot be their friends. Furthermore, professionals themselves also ought to realize they were clients' caregivers and not their friends, faculty said. So here again the instrument strengthened the problematisation, and led to a situation that faculty of the project liked to see: clients placing professionals where they belong.

So although clients' wishes had to be leading in the improvement actions, their perspective was not taken for granted. Perceiving professionals to be friends was thought to be problematic. Some teams, therefore, were struggling with adapting improvement actions to their clients' wishes but at the same time had to confront clients with a picture of reality that was not the way clients perceived it. In this sense, the problematisation of faculty both strengthened and denied the perspective and wishes of some clients.

#### The effects of the instrument

Apart from strengthening faculty's problematisation, the instrument may have other effects. For one, it could heighten clients' awareness of their social networks. One client reported forty contacts at the start of the project, a number reduced to no more than twenty at the end. Faculty thought this might be due to more awareness of what really could be regarded as friends, and mentioned this awareness in general to be one of the successes of the project. For that matter, a project leader pointed out that the visual nature of the instrument makes the social situation of clients clear at a glance. Therefore clients could easily replace and relocate contacts. One client for example found out that a perceived friend was actually not a friend, and vice versa.

In some cases clients' heightened awareness of their social networks made improvement actions superfluous. For example, a client who always said he was very lonely was astonished to see how big his network was and how active he was. "Why complain about being lonely at all", he was reported to say. So the use of the network circle instrument led clients "to redefine the concept of loneliness", as this project leader said. Apart from its positive effects, however, the instrument could evoke more negative feelings when clients were confronted with their small networks.

In the above examples the instrument transformed the way in which the actors involved thought about and enacted the improvement situation, and their social life in general. These transformations may have been foreseen. Still, as a possible unforeseen side-effect, use of the instrument often improved relations between professionals and clients as well. Even clients who did not gain any new contacts enjoyed talking about their social networks, a project leader said. All this, however, was rather not in line with faculty's policy of strengthening the inner network circles instead of the outer ring consisting of professionals.

#### Analysis of the measurement practices

To summarize, in the 'social participation' project the measurement instrument selected by faculty had several roles. It not only measured results, but also steered improvement actions in the desired direction. Faculty's problematisation was that professionals tended to be unaware of what clients would like and also did not ask them. Moreover, clients themselves sometimes lacked awareness of their social situation. Having them to 'objectively' classify their relations was thought to be a solution for this shortcoming.

Furthermore, the instrument assumed a typical client, one willing and able to discuss social relations with professionals who could not be conceived as friends anymore. Faculty assured that clients had an active role; they needed to think about what they would like and discuss this with care givers. The instrument thus had a performative effect; i.e. it shaped reality as well [21]. As this example illustrated, measurement practices in improvement projects not only endorse faculty's problematisation but also carry (subtle) assumptions about who should be able and who has the obligation to do something about the problem. Measurement practices may change the improvement practices in foreseen and unforeseen ways.

## Conclusion

In this article we proposed a way for opening the black box of QIC's, going beyond a mere description of the elements or the extent to which they are implemented. We studied a collaborative in action and analyzed how it was formed over the course of one-year-long improvement projects. To illustrate our method and to actually open the black box we zoomed in at the problematisation process and the related measurement practices. Our empirical material came from two projects in a larger collaborative aimed at improving mental health care and care for the intellectually disabled.

The problematisation process in the 'recovery-oriented care' project proved to have undergone a transformation. At baseline professionals mainly sought to support clients' recovery process by not hindering it. Later on many teams were trying to stimulate clients. This problematisation process also had consequences for the different roles proposed for the actors involved; it had consequences for "who has the right and who has the obligation" to do something about the problem [27]. So by using an ANT-perspective we showed that the topic is not fixed and given prior to the collaborative, but instead is formed within the collaborative, both by the expert knowledge of faculty and the local knowledge of improvement teams.

This change in problematisation may have been more pronounced in the projects we studied because the improvement teams were free to come up with their own targets, to 'do' their own problematisation. This may be different for other improvement projects. When improvement teams have that much leeway, a dynamic perspective is even more needed if only to see what the improvement project is all about.

To further open up the black box, we studied the role of the measurement practices within the 'social participation' project. As effectiveness studies often assume a direct relation between a topic and its measurability, researchers should unravel this relation, as we suggested. Our analysis showed that the measurement instrument is linked to the problematisation in more than one way. First, it measures the effectiveness of the collaborative in reaching the predefined goal(s). Secondly, it may strengthen the problematisation, supporting both the problem and the solution that faculty proposed. The instruments then may make it more likely that the teams will adopt the problematisation. But moreover, there were effects on the improvement practices as well. Measurement instruments inevitably carry assumptions, for example that clients are willing and able to have conversations about their network, and would do well not to count professionals among their friends. Therefore measurement instruments also co-define and co-produce the actors involved, and thus have a performative effect on the practices they measure [21].

Both human and non-human actors play a role in constructing the collaborative, as we showed by using an ANT-perspective. However, by using this perspective, we were less concerned with the "why" or the intentionality question [28], for example with the question why the project was framed in a certain way or why the faculty team of the 'social participation' project urged professionals and clients alike not to classify professionals as clients' friends. Instead we focused on the performance of a project and what consequences this performance has for the actors involved.

By focusing on these questions, we showed that the problem cannot be assumed to stay fixed over the course of the project. Yet these changes in problematisation do not automatically have consequences for measuring the effectiveness of the collaborative. In the 'recovery-oriented care' project, the solution changed, but teams were still trying to solve the same problem that faculty pointed at: the lack of future perspective. It depends on the actual goal of the program and on the indicators for success if changes imply a change in effectiveness as well.

But as much "summative" research assumes that goals do not change during the project, it is important to test this assumption. Otherwise, it is hard to ascribe the effectiveness - or lack of effectiveness - to the improvement actions and the collaborative method. So it would seem crucial not only to report on outcomes but also to analyze what happened in the collaborative. Therefore, our analysis can be seen as a plea for a mixed methods approach. This mixed methods approach is part of an ongoing debate and although some scholars argue for such an approach, the extent to which it is actually done leaves much to desire [3,4].

Bate and Robert in contrast argue that a summative evaluation (mainly quantitatively) and a formative evaluation (mainly qualitatively) will not mix at the end of the day: "Although there are some overlaps and similarities, they are, in our view, ultimately incompatible and incommensurable research paradigms (...)" [5]. A formative approach implies intervening in the object one studies, which affects the outcomes, they say. This creates "impossible and, largely, unmanageable tensions" between intervention and experiment for no valid statements can be made about the method itself affecting certain results [5].

Yet our analysis can be read as a contradiction of, or at least as a critical note to, their argument. We have seen that measuring practices indeed can have a performative function. These measurement practices of improvement teams themselves are often used by evaluation researchers to examine effectiveness of QIC's. Therefore, this type of evaluation research has to deal with the same performativity of the measurement practices, which has consequences for the proposed distinction between intervention and experiment. But even if measurement instruments are used that are not owned by improvement teams themselves, one could question if these are free from performative effects. Even the fact that measuring takes place already influences its outcomes and thus can be seen as an intervention in itself [16,21,22].

Yet the performative effect of measuring will only occur, or will be more likely to occur, if measuring is part of a reflexive process [29]. If not reflected upon it cannot lead to more awareness of a situation, it cannot lead to redefining of concepts or to confrontations with certain circumstances, which may all be effects of measuring as we have seen in the analysis. So only if this reflection occurs - which is highly likely when questions are asked to clients, as in our case - measuring is a performative process.

When accepting that measuring - given reflexivity - can have (per)formative elements as well, there will be no unmanageable tensions, no incommensurable paradigms between intervention and experiment. Measuring is based on an interpretative and supposedly also a performative process which is thus a formative instrument in itself. If there are always tensions between intervention and experiment, this tension is no valid reason for not combining the more formative and the more summative research. Besides, as collaboratives are governmental instruments to improve certain aspects of care, they deserve confirmation of the expected outcomes. Thus there is every reason to explore ways to intelligently combine qualitative and quantitative methods, gaining insight both into the outcomes and in the (dynamic) construction of the collaborative. This then is the next challenge for evaluation researchers of quality improvement collaboratives.

## Competing interests

The authors declare that they have no competing interests.

## Authors' contributions

TB collected ethnographic data collection at the conferences and within the organizations. AN and RB helped to draft the manuscript and took part in reviewing it. All authors read and approved the final manuscript.

## Pre-publication history

The pre-publication history for this paper can be accessed here:

http://www.biomedcentral.com/1472-6963/10/265/prepub
